# Microvesicle-mediated release of soluble LH/hCG receptor (LHCGR) from transfected cells and placenta explants

**DOI:** 10.1186/1477-7827-9-64

**Published:** 2011-05-15

**Authors:** Anne E Chambers, Paul F Stanley, Harpal Randeva, Subhasis Banerjee

**Affiliations:** 1 Department of Clinical Biochemistry, Laboratory Medicine, Birmingham Heartlands Hospital, Bordesley Green East, Birmingham B9 5SS, UK; 2 Centre for Electron Microscopy Metallurgy and Materials Building, University of Birmingham, Edgbaston, Birmingham B15 2TT, UK; 3 Clinical Sciences Research Institute, Medical School Building, Gibbet Hill Campus, University of Warwick, Coventry, UK

## Abstract

Placental hCG and pitutary LH transduce signals in target tissues through a common receptor (LHCGR). We demonstrate that recombinant LHCGR proteins which include the hormone-binding domain are secreted from transfected cells and that natural LHCGR is also secreted from human placental explants. LHCGR recombinant proteins representing varying lengths of the N-terminal extracellular domain were expressed in Chinese Hamster Ovary cells in suspension culture. Secretion was minimal up to 72h but by 96h 24-37% of the LHCGR had been released into the culture medium. The secreted proteins were folded and sensitive to glycosidases suggesting N-linked glycosylation. Secretion was independent of recombinant size and was mediated via structurally defined membrane vesicles (50-150nm). Similarly cultured human early pregnancy placental explants also released LHCGR via microvesicles. These studies provide the first experimental evidence of the possible mechanistic basis of the secretion of LHCGR.

## Background

Reproductive hormones (luteinizing hormone [LH], follicle stimulating hormone [FSH] and human chorionic gonadotropin [hCG)]) are collectively known as gonadotropins because they stimulate gonads (testes in male and ovaries in female). The hormonal activation occurs through specific ligand-receptor interactions on the surface of the target cells. The LH and hCG utilize a common receptor encoded by a single copy ~70 kb *LHCGR *gene, located at human chromosome 2p21 [1]. *LHCGR *has 11 exons and codes for multiple alternatively spliced species (at least 6) of mRNA. Transcriptional activation to generate these mRNAs is initiated at multiple sites spanning a region more than a kilobase upstream of the first exon [2]. Alternatively spliced mRNAs produce several truncated proteins which have the ligand binding capacity but are ineffective in transducing signals [3-7]. In addition to testis, ovary and placenta, various isoforms of LHCGR are expressed in uterine myometrium, vascular endothelial and smooth muscle cells, adrenals, brain, skin, lymphocytes, human sperm, macrophages and in fetal tissues [[8], for a review].

The earliest biochemical evidence on the existence of cell-free soluble LH receptor was the purification of an hCG-binding protein, relative molecular mass of 65K (M_r_, 65K) from porcine follicular fluid and was based on gel filtration followed by affinity chromatography [9]. A different experimental approach by West and Cooke [10] demonstrated the secretion of M_r _80-90K hCG-LH receptor complex into the culture media from ligand-induced rat and mouse leydig cells. Tsai-Morris *et al*. [11] first reported the production and secretion of soluble LH receptor following transient transfection of a naturally truncated variant of the receptor in COS cells. These data were independently substantiated using different combinations of *in vitro *expression systems [12,13], In some studies, the lack of detection of an actively secreted soluble form of expressed LH receptor was attributed to misfolding and intra-cellular retention of the expressed protein or proteolytic degradation of the protein released into the culture media [13,14]. Moreover, the lack of a secreted form of the receptor was overcome by co-expression of hCG with the extra-cellular domain of porcine LH receptor leading to the secretion of a mixture of free as well as hCG-receptor complex [13].

Notably, the natural rat Lhcgr variant first described by Tsai-Morris *et al*. [11], instead of being secreted, was retained in the ER prior to degradation [15]. Additionally, the truncated Lhcgr, misrouted the full-length Lhcgr in ER, thereby decreasing the cell surface expression of the mature functional receptor. The role of circulating sLHCGR as in inhibitor of LH/hCG functions by ligand binding has been questioned; instead, a specific intra-cellular role of truncated receptor in regulating the cell surface expression of a full-length ligand-binding Lhcgr has been proposed [15]. Therefore, how a complex LHCGR protein, embedded within the membrane lipid bilayer, could be secreted as soluble receptor remained unexplained.

Structurally, LHCGR is similar to thyroid and follicle stimulating hormone receptors (TSHR and FSHR respectively). The autoantibody-binding extracellular domain (ECD; N-terminal 418 amino acid residues) of TSHR when expressed in CHO cells had a high content of mannose residues, was misfolded and retained in cells. However, expression of progressively truncated carboxy- terminal domains of TSHR in stably transfected CHO cells resulted in the secretion of the TSHR ECD with an efficiency that was inversely proportional to the length of the recombinant proteins [16]. These reports prompted us to generate C-terminally truncated recombinant variants of LHCGR and examine the expression of the secreted recombinant proteins in serum-free medium. Contrary to our expectation, we have demonstrated here that the release of soluble LHCGR protein from transfected cells as well as from placental explants was independent of the length of the receptor protein and mediated via structurally defined membrane vesicles.

## Methods

### Construction of LHCGR cDNA clones

Initially, three LHCGR cDNA clones inserted into the mammalian expression vectors (30233-0, 30233-1, 30233-2) were created. A cDNA fragment encoding the N-terminal 318 amino acids of human LHCGR (CCDS1842.1; UniProtKB/Swiss-Prot P22888) was obtained by polymerase chain reaction (5'-AAGCTTATGAAACAGCGTTT-3' and 5'-GGATCCCAGGGTCTTGTTAC-3'; forward and reverse primers containing *Hind*III and *Bam*HI sites respectively). The PCR amplified fragment was engineered to have a CTGCAG insertion (corresponding to Leu-Gln, LQ insertion) at nucleotide position 55-60 (amino acid residues 19-20). The idea behind this being that the LQ containing polymorphic variant in the 'signal peptide' appears to express and function efficiently (2, 4). The resultant 966bp PCR amplified fragment (30233-0) was cloned into the HindII and BamHI site of p3XFLAG-CMV-14 vector (Sigma Aldrich). The cDNA encoding the first 291 amino acids of LHCGR (30233-1) was created employing 885 bp PCR fragment (5'-AAGCTTATGAAACAGCGTTT-3' and 5'-GGATCCCTCTTTAGTGGGCA-3', forward and reverse primers respectively). The cDNA encoding the first 229 amino acid residues of LHCHR (30233-2) was obtained by PCR amplification of 699bp fragment (5'-AAGCTTATGAAACAGCGTTT-3' and 5'-GGATCCGAGGGTCTTGGGGC-3', forward and reverse primers, respectively). To generate 30233-3 (a vector containing the N-terminal 416 amino acids of LHCGR), a 300bp cDNA (5'-TACTCCTCCA-3' and 5'-GGCGATCAGC-3', forward and reverse primers, respectively) was ligated to the 3' end (corresponding to the C-terminus) of 30233-0. The codon optimized DNA sequences of each clone were verified prior to plasmid amplification and purification. Plasmid DNA was prepared using Purelink HiPure Plasmid kit (Invitrogen) and sterile filtered through a 0.22 μm filter.

### Cell culture, transfection and expression of recombinant LHCGR protein in CHO-S cells

One vial of 10^7 ^frozen CHO-S cells was added to 30 ml of pre-warmed serum-free Freestyle CHO medium (Invitrogen) containing 8 mM L-glutamine without antibiotics in a 125 ml disposable polycarbonate spinner flask with vented cap (Sigma-Aldrich) and incubated at 37°C, 8% CO_2 _with 150 rpm stirring. Total cell count and cell viability were monitored every 24h. A cell viability of >70% was required in order to continue with the cell expansion. Cells were subcultured by seeding at a cell density of 0.3 - 0.5 × 10^6 ^viable cells/ml. The cell density was never allowed to exceed 2 × 10^6 ^cells/ml. Following expansion, cells were pooled for measurement of cell count and viability. The cell viability of expanded cells before transfection exceeded 95%. Pooled cells were used to seed fresh flasks of pre-warmed serum free medium containing 8 mM L-glutamine at a density of 1 × 10^6 ^cells/ml in a minimum of 60 ml per 125 ml spinner flask prior to DNA transfection.

75 μg (75 μl) of filter sterilized plasmid DNA was used for each transfection of 1 × 10^6 ^cells per ml in a total volume of 60 ml of cells. The sterile DNA was first diluted into Opti-Pro SFM (Invitrogen) to a total volume of 1.2 ml and mixed. In a separate tube, 75 μl of FreeStyle Max Transfection Reagent (Invitrogen) was added to Opti-Pro SFM to a total volume of 1.2 ml and mixed gently by inverting the tube. The diluted FreeSTyle MAX Transfection Reagent was added to the diluted DNA solution to obtain a total volume of 2.4 ml and mixed gently. As a negative control (no DNA and no FreeStyle MAX Reagent), 1.2 ml of Opti-Pro SFM was mixed with 1.2 ml tissue culture medium. After incubation at room temperature for 10 minutes, the DNA-FreeStyle MAX mix was added to the CHO suspension culture and the flasks were transferred to the incubator (37°C, 150 rpm, 8% CO_2_). Following transfection, aliquots (4ml) was removed from each transfection every 24h up to day 7 (D7). No extra tissue culture medium or passaging of cells was carried out after transfection. Both cell density and cell viability for each culture were recorded. Aliquots were collected in sterile 15 ml conical tubes and spun at 700 rpm in a Beckman J6 centrifuge using a JS-4.2A rotor for 6 minutes at room temperature. To monitor any secretion of proteins from the cells, the tissue culture medium (supernatant) was carefully removed to another sterile tube after centrifugation, leaving the last 200 μl so as not to disturb the cell pellet. Supernatents were stored at -20°C. Cell pellets were washed three times with sterile PBS (4°C) to remove residual tissue culture medium and transfection reagents, aspirated and stored at -20°C.

### Placenta explant culture

This study was approved by the local ethics committee of Planned Parenthood, Fannin Health center, Houston, Texas and written consent was obtained from patients before the collection of samples. Placental tissue was obtained from patients undergoing elective termination of pregnancy with gestational age range of 9-12 wks (gestational age was determined by ultrasound measurement of biperietal diameter or crown-rump length). Immediately after collection, the placenta tissue was rinsed with sterile PBS and sequentially washed (10-12 times) to remove traces of blood. The tissue was dissected in a sterile cell culture hood to produce small pieces of placental explants (20-50 mg in weight, approximately 0.5-1.5 mm) and was resuspended in serum-free culture medium. The suspension was transferred to disposable sterile spinner flask and was cultured at constant stirring speed 150 rpm under 8% CO2 up to 48h.

### Protein extraction, Western blots and quantitative analysis of protein expression

Protein extraction from cultured cells was carried out using hypotonic buffer (10 mM Tris-HCl, pH7.5 containing 25 mM EDTA, 25 mM, NaF, 1 mM Na_3_VO_4_, and EDTA-free protease inhibitor mix [Sigma-Aldrich]). Detergent extraction of cell culture pellets following hypotonic lysis was carried out with lysis buffer (25 mM Hepes pH 7.5, 150 mM NaCl, 1% igepal CA-630 [Sigma-Aldrich], 10 mM MgCl_2_, 1 mM EDTA, 25 mM NaF, 1 mM Na_3_VO_4_, and EDTA-free protease inhibitor mix [Sigma-Aldrich]. This sequential extraction method was employed to obtain a predominantly cytosolic extraction (via hypotonic lysis) and a predominantly membrane extraction (hypotonic followed by detergent lysis). Extraction of placental villous tissue was performed using either T-PER reagent alone [Perbio, Helsinborg, Sweden] or T-Per containing 25 mM bicine buffer pH 7.6 [Thermo Fisher Scientific].

Western blot analysis was as described [17,18]. Primary and secondary antibodies used were as follows: anti-human LHCGR mouse monoclonal antibody [LHR-29, ATCC clone CRL2685]; anti-FLAG monoclonal antibody [Sigma]; anti -β Actin, clone AC-15 [Sigma] and goat anti-mouse IgG (H+L) HRP-conjugated [Chemicon International Inc., CA, USA]; mouse control IgG [Sigma]. The antibody concentrations used for Western blots were: LHR-29 diluted to 1 μg/ml; anti-FLAG at 1:1000 dilution and anti-mouse IgG-HRP at 1:10,000 in the incubation medium. The epitope which the LHR29 monoclonal antibody recognizes has been mapped to a region between amino acids 227 to 289 of the LHCGR ECD (unpublished data).

### Protein deglycosylation

Five times concentrated N-Glycanase Reaction Buffer (100 mM sodium phosphate pH 7.5, 0.1% sodium azide); Denaturation solution (2% SDS, 1 M β-mercaptoethanol); Detergent Solution (15% NP-40 solution); Endo H and PNGase F (glycerol-free) were obtained from Prozyme [San Leandro, California, USA]. According to vendor's protocol, both Endo H and PNGase F were added to a final concentration 50 mU/ml and 50 U/ml, respectively. Samples were incubated at 30°C for 16 h and reactions were terminated by adding SDS-PAGE loading buffer.

### Microvesicle purification from culture supernatants

The placenta explant cultures were collected at 24h and 48h. The explant tissues and the cell debris were first pelleted by centrifugation at 3000 × g for 15 min. The supernatant was re-centrifuged at 14000 × g for 15 min and two-thirds of the clear supernatant from the top of each tube was transferred to new tubes prior to the purification of microvesicles by two alternative methods; ultrafiltration [19] or ultracentrifugation [20,21]. For ultrafiltration, the vivaspin nanomembrane concentrators [Vivasin 500 Sartorius Inc., Goettingen, Germany] were washed twice with 600 μl PBS (with centrifugation at 300 × g) for 5 min to remove glycerol and other preservatives. Subsequently, 600 μl of the clear culture supernatant was centrifuged in each concentrator column at 6000 × g for 10 min at 4°C. This process was repeated and the combined retanate was then mixed with EDTA-free protease inhibitor mix (Sigma Aldrich) and stored at -20°C. Alternatively the culture supernatant was centrifuged at 10000 × g [L8-70M ultracentrifuge, Beckman Coulter, Inc. CA; 70.1 Ti rotor] for 1h and the pellet was resuspended in PBS containing EDTA-free protease inhibitor mix ]Sigma-Aldrich] before storage at -20°C.

### Staining of microvesicles and immune-electron microscopy

For negative staining, grids were incubated at room temperature for 10 min with 25 μl of 2% uranyl acetate and examined by electron microscopy [Jeol 1200EX TEM operating at an acceleration voltage of 80 kV]. For immuno-labelling, 10 μl of microvesicles suspension was dropped onto a 200 mesh nickel Formvar/Carbon coated grid [Agar Scientific-S162N] and incubated for 40 min in a humid box at room temperature. A drop of 2% paraformaldehyde (PFA) in 0.1 M phosphate buffer, pH7.2 was added to each grid. Following PFA, grids were treated sequentially with 0.15% glycine, 1% BSA in phosphate buffer followed by primary antibodies (anti-LHCGR, LHR29 or anti-FLAG monoclonal antibodies or mouse IgG, diluted 1:10 in phosphate buffer with 1% BSA) for 30 min at room temperature. Unbound primary antibodies were removed by rinsing with 0.1% BSA in phosphate buffer four times, 2 min each time. The grids were labelled with secondary antibody conjugated with 10 nm colloidal gold [Clone M2, Sigma-Aldrich] for 1h at room temperature in a humid box and then washed multiple times with phosphate buffer before fixation using 1% glutaraldehyde in 0.1 M phosphate buffer for 5 min at room temperature. Following removal of excess glutaraldehyde, grids were washed six times with distilled water for 1 min each time prior to negative staining with 2% aqueous uranyl acetate for 1 min. All experiments were done in duplicate. Samples were examined in a Jeol 1200EX transmission electron microscope and images were captured and stored.

### Densitometry and data analysis

Densitometry of autoradiograms and data analysis were carried out as described [17,18]. In the majority of cases the analyses used β-actin as an internal control for computing the experimental values. However, in some experiments (see 'Results' below), where the β-actin failed to reflect the yield of LHCGR in the supernatant fraction, a M_r _63K membrane protein, stained using Coomassie blue following Western blot, was used as an internal standard to measure the corresponding LHCGR protein. A value for the level of significance (*P*-value) was calculated using the Poisson statistic. *P *< 0.05 was considered significant.

## Results

### Expression of LHCGR recombinant proteins in CHO cell suspension culture

**Figure 1:**
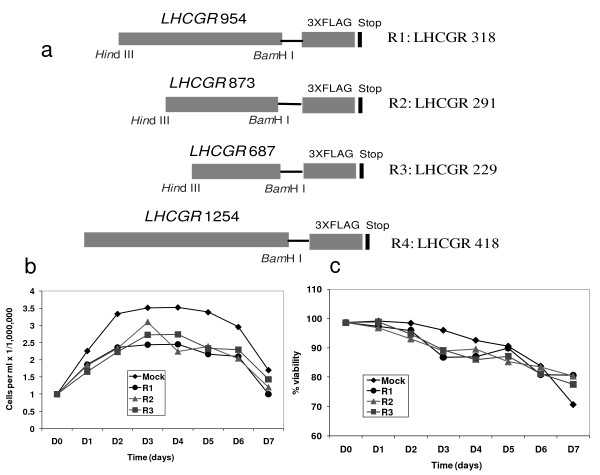
**The LHCGR recombinants and expression in CHO cells**. a), The LHCGR cDNA recombinants used for *in vitro *expression of the receptor protein in CHO cells grown in suspension. The C-terminus of each recombinant contained FLAG tag; b), the cell densities through 7 days of transfection with plasmids R1: LHCGR-318, R2: LHCGR-291 and R3: LHCGR-229 as well as the control (transfection reagents alone). Day 0 is the start of transfection while days 1-7 are post-transfection; c), the cell viability expressed as a percentage of total cell count in plasmid transfected and control cultures through 8 days of the experiment.

Chinese hamster ovary suspension (CHO-S) cells grown in serum-free medium at a cell density of 10^6 ^cells per ml were transfected with mock or with one of three LHCGR recombinants; LHCGR-229, LHCGR-291, LHCGR-318 (Figure 1a) and the cell density and cell viability were recorded for up to seven days post-transfection. The recombinant LHCGR- 418 has a part of the first trans-membrane domain of the receptor (Figure 1a) and was transfected separately in subsequent experiments (data not shown). Exponential cell growth resulted in a cell density of 3.5 × 10^6^/ml and 2.4-3.1 × 10^6^/ml in mock and DNA transfected cultures, respectively, at day three (D3), remained stable until day four (D4) and began to deccline at day five (D5) (Figure 1b). Notably, the cell density dropped significantly on D7 compared to that on D3 in mock (*P *= 0.02), recombinants R1- (*P *= 0.021), R2- (*P *= 0.007) and R3- (*P *= 0.038) transfected cells. Unlike the cell density, the cell viability was highest on D1, and mean cell viability decreased gradually; D3 (90.2%), D4 (88.7%), D5 (88.2%), D6 (88.2%) and D7 (77.2%). However the decrease in cell viability during the course of the experiment was not significant (*P *≥ 0.10 on D7).

In order to examine the expression of recombinant LHCGR expression, samples were aliquoted from each transfection including corresponding control cultures at 24-hr intervals for up to seven days (D7). To distinguish the cytoplasmic soluble LHCGR (sLHCGR) from the cell membrane-associated receptor protein, the cells were first lysed in hypotonic buffer. Following hypotonic extraction, the cell pellet was subject to equal volume of detergent (Triton) lysis to recover the membrane-bound LHCGR recombinants. The cumulative expression at each time point for each recombinant was expressed as sum of the products in hypotonic and detergent lysates.

**Figure 2:**
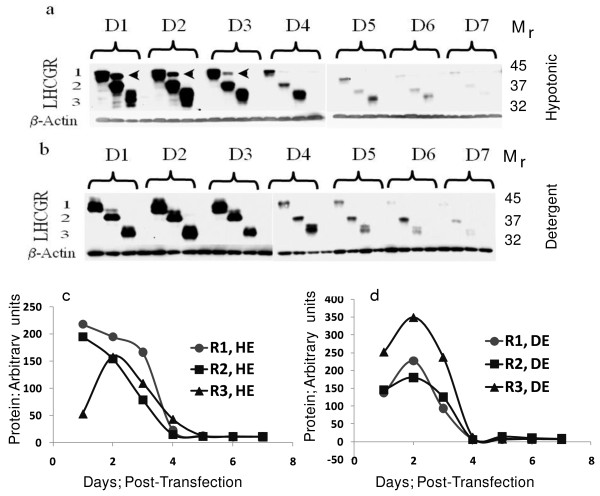
**Time course analysis of the expression of the LHCGR recombinants**. Three recombinant LHCGR proteins expressed in a), the cytosolic fractions (hypotonic lysis) and b), those associated with the cell membrane (sequential hypotonic and detergent lysis). Equal amounts of protein for each 24h time-point (D1 to D7) and each recombinant (R1, R2 and R3) were resolved by 8% SDS-PAGE and, following transfer of the proteins via Western blotting, were first probed with anti-FLAG monoclonal antibody and subsequently with a β-actin specific monoclonal antibody. The estimated molecular mass (M_r_) of recombinants 1, 2 and 3 were 45K, 37K and 32K, respectively. LHCGR number 1, 2 and 3 represent recombinants R1, R2 and R3 as shown in Figure 1. The density of each band was measured, normalized to the corresponding β-actin value and the expression of each LHCGR recombinant in c), cytosolic (hypotonic) and d), membrane-bound (detergent) fractions were plotted to evaluate the quantitative expression.

The most obvious results from these experiments were that all three recombinants were expressed at high levels until 72h following transfection (D3) and there was a dramatic reduction in expression on D4. Quantitatively, cytoplamic sLHCGR expression for R1-318, R2-291 and R3-229 on D4 was reduced by 7.3-, 5.3- and 2.5-fold respectively compared to that on D3 post-transfection (Figure 2a and 2c). Similarly, analysis of the membrane-associated (detergent lysis) LHCGR showed that the production from R1-318, R2-291 and R3-229 on D4, compared to D3, was reduced by 13-, 21- and 18.8-fold, respectively (Figure 2b and 2d). Moreover, when analysis was extended to the cumulative (hypotonic plus detergent) LHCGR expression, the R1-318, R2-291 and R3-229 on D4, compared to D3, was reduced by 8.6-, 9.9- and 6.2-fold, respectively. Taken together, these results demonstrate that the LHCGR recombinant proteins were synthesized abundantly during the first three days following transfection and that the production or accumulation was signifiantly down regulated from D4 to D7.

### Secretion of sLHCGR into the culture medium

**Figure 3:**
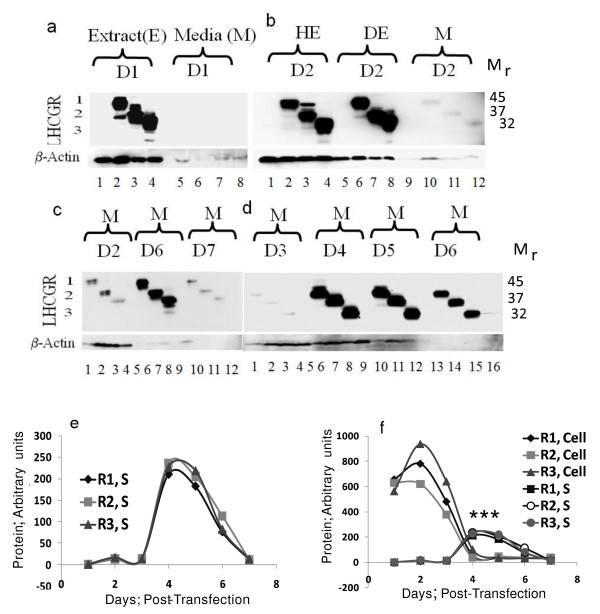
**Secretion of LHCGR recombinant proteins**. Cell culture supernatants (M) at a), 24h and b), 48h following mock transfection or transfection with LHCGR cDNAs were centrifuged and concentrated, and corresponding whole cell extract (E), hypotonic (HE) and detergent (DE) extract proteins were resolved via SDS-PAGE before detection by anti-FLAG antibody in Western blots. Tissue culture supernatants (M) prepared from c), 48h, 144h and 168h following transfection; the results shown in d), demonstrate abrupt increase in secretion of the recombinant LHCGR on D4 (96h) following transfection; e) and f), quantitative analysis of secreted proteins through seven days post-transfection showing the relative amounts of each secreted recombinant protein compared to cytosolic plus membrane associated recombinant protein. The cell-associated and the supernatant LHCGR values were derived by comparing the expression of each recombinant protein from the same culture volume (Figure 3f). LHCGR number 1, 2 and 3 represent recombinants R1, R2 and R3 as shown in Figure 1. The concentration of the LHCGR recombinants in the supernatant was highest on day 4. The mean expression of R1, S (*P *< 0.001, R2, S (*P *< 0.001) and R3, S (*P *< 0.0005) was significantly higher (indicated by ***) compared to that of corresponding cell-associated LHCGR on D4 and D5.

The results shown in Figure 2 demonstrated that irrespective of the length of the recombinant protein, the maximal expression of the LHCGR recombinants occurred within first 48h post-transfection. A logical extension of these data is that should there be any secretion of these proteins into the culture medium, it might be detected by analysing the corresponding culture supernatants. To address this, the cell culture supernatants from mock and experimental samples at 24h and 48h were concentrated by ultra-filtration (see Materials & methods) and analysed by resolving the proteins via polyacrylamide gel electrophoresis under denaturing conditions (SDS-PAGE). The data shown in Figure 3a demonstrate that while no LHCGR could be detected at 24h, all three recombinants were barely detectable in culture supernatants taken at 48h post-transfection (Figure 3b). The lack of signal in 24h supernatant was unexpected because with the exception of LHCGR-229 (*P *= 0.001), the cumulative cellular expression (hypotonic plus detergent) of LHCGR-318 (*P *= 0.15) and LHCGR-291 (*P *= 0.58) at 24h were not significantly different from those at 48h. To examine whether longer incubation introduced cellular stress resulting in the secretion of sLHCGR, the culture supernatants from D2, D6 and D7 were analysed. The data presented in Figure 3c show that, contrary to our expectation, the highest secretion of sLHCGR was at D6 and the secretion at D2 and D7 was comparable. Further analysis of the culture supernatants revealed that sLHCGR secretion reached a peak on D4 and remained high until D6 (Figure 3a-d).

The amount of secreted recombinant LHCGR-318, LHCGR-291 and LHCGR-229 at D4 (Figure 3b-d and 3e), was 26.9%, 37.4% and 24.2% respectively (Figure 3f), compared to their highest cumulative cellular expression on D1 or D2 (Figure 2). Additionally, the amount of LHCGR-318, LHCGR-291 and LHCGR-229 in the culture supernatant at D4 was 7-, 11.5- and 4-fold, respectively, when compared with corresponding cellular expression on D4 (Figure 2 and Figure 3e and 3f). Similar results were obtained where secretion of LHCGR-418 into the medium was maximum on D4 (data not shown). The cellular expression of the recombinants was highest on D2 and subsequently declined. And yet, the amount of protein secreted on D4-D6 was significantly higher than the total protein produced by the cell (intracellular) on D4-D6 (Figure 3f). This paradoxical observation could be explained assuming that the cellular microvesicles accumulated up to D3, were abruptly released into the media between D3 and D4. Once released, the cellular production of the recombinant protein was almost shut down and the microvesicle-bound LHCGR proteins underwent gradual proteolytic degradation on D5 and D6 before disappearing on D7. Interestingly, the cell density being high on D3 and D4, began declining afterwards (Figure 1b).

### Secreted LHCGR proteins are glycosylated and folded

**Figure 4:**
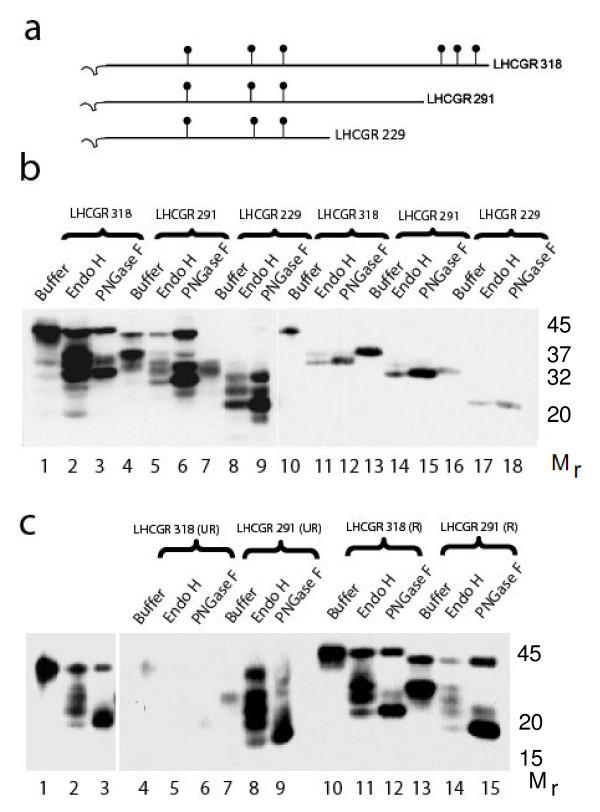
**Patterns of glycosylation of cell-associated and secreted LHCGR recombinant proteins**. a), the relative position of the glycosylation sites in the three recombinants; b), the LHCGR 229-318 residue proteins from transfected cell extracts (lane 1-9) as well as concentrated culture supernatants (lanes 10-18) at 96h following transfection were digested with Endo-H and PNGaseF prior to Western blot analysis as described; notably the protein isoforms resistant to deglycosylation by both enzymes were not secreted; c), both unglycosylated and glycosylated LHCGR 291 and LHCGR 318 migrate faster under non-reducing (UR) compared to reducing (R) conditions suggesting that folding of the proteins is independent of glycosylation. Lanes 1-3 are longer exposure of lanes 4-6.

The level of glycosylation of recombinant LHCGR proteins was examined by *in vitro *deglycosylation with peptide N-glycosidase F (PNGase F) and endopeptidase H (Endo H). The PNGase F, an amine oxidase, cleaves between asparagine and N-acetylglucosamine (GlcNac) releasing high mannose, hybrid and complex oligosaccharides from N-linked glycoproteins. Therefore, PNGase has the capacity to remove all N-linked oligosaccharides from glycoproteins. EndoH primarily releases high mannose-type from N-linked glycoproteins by cleaving between two core GlcNac of high mannose chains. The recombinant LHCGR-318 has six glycosylation sites whereas LHCGR-291 and LHCGR-229 proteins each have three sites (Figure 4a). All three recombinants were fully glycosylated in CHO cells and were susceptible to both PNGase F and EndoH (Figure 4b). However, a fraction of LHCGR-318 and LHCGR-291 were resistant to both enzymes. Notably, these non-glycosylated (glycosidase-resistant) species of LHCGR-318 and LHCGR-291 were released from cells by hypotonic lysis indicating that they are soluble cyoplasmic proteins (indicated by arrows in Figure 2a). Moreover, these proteins, despite being soluble were not secreted into the media (Figure 2a and Figure 3b-d). We conclude that the majority of the recombinant LHCGR was glycosylated and that this fraction, irrespective of membrane or cytoplasmic association, was secreted from the cells, whereas, a minor fraction of the recombinant protein devoid of N-linked glycans, was not released into culture medium. Irrespective of the level of glycosylation, the recombinant LHCGR proteins migrated faster under non-reducing conditions compared to reducing conditions when disulphide bonds were broken (Figure 4c) suggesting that folding of the recombinant LHCGR was independent of glycosylation.

### Selective release of proteins into the culture medium

**Figure 5:**
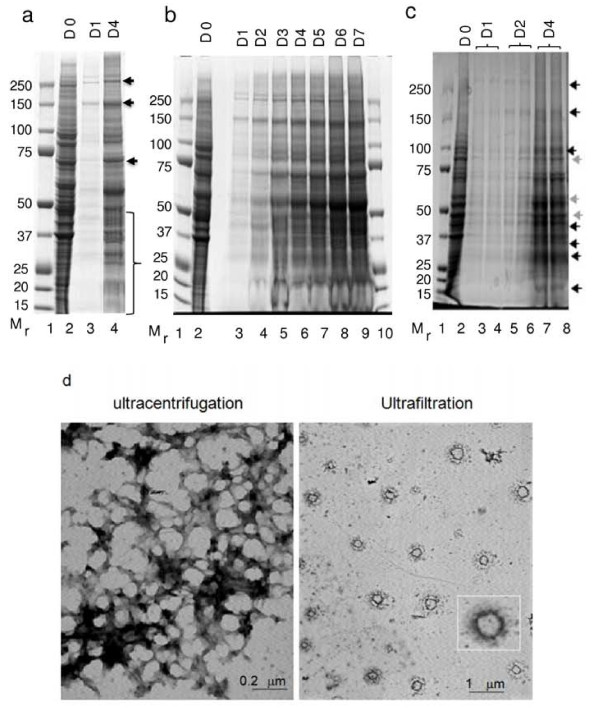
**Selective secretion of proteins from transfected cells through microvesicles**. a), the whole cell extracts (lane 2, Cont), the culture supernatants (D1 and D4) following transfection with LHCGR 291 cDNA were centrifuged at low and high speed to remove the cell debris and subsequently concentrated prior to electrophoresis in 4-12% Bis-Tris polyacrylamide gels; b), same as a) except the medium concentrated from LHCGR 291 cDNA transfection from D1-D7 are shown; c), shows that proteins secreted from the mock transfected as well as cDNA transfected cells are similar, the dark and light arrows indicate quantitative enrichment and reduction of proteins secreted in the medium respectively; d), electron micrographs of D4 culture supernatents concentrated by ultracentrifugation (left panel) and by ultrafiltration (right panel). The microvesicle shown in the inset is a 12-fold magnification of that seen at the right-bottom corner of the figure, below the inset.

Three critical observations prompted further investigation on the secretion of LHCGR into the culture medium; The abrupt release of sLHCGR on day 4 post transfection when cellular synthesis of the proteins reduced significantly (Figure 2c and 2d); failure to detect the cytoplasmic sLHCGR species that were resistant to deglycosylation in the culture medium (Figures 3 and 4) and quantitative reduction of β-actin secretion in the supernatant (Figure 3a-d) which suggested selective secretion of the cellular proteins (Figure 3). To investigate these observations, the protein composition of the cell culture supernatant at D1 and D4 was first compared with that of untransfected CHO cells. The data shown in Figure 5a, indicate that a large number of proteins (ranging from M_r _10K to 250K) were selectively lost or enriched in the supernatant (Figure 5a). A further investigation comparing the supernatant fractions from D1-D7 with their respective total cellular proteins revealed that the differential release of cellular proteins into the culture supernatant began on D1 and continued to increase until D7 (Figure 5b). The resolution of proteins from the culture medium through an alternative gel system (Figure 5c) showed that some proteins were enriched (indicated by dark arrows) while others were depleted (indicated by light arrows) in the supernatant. This selective secretion of the proteins was independent of the recombinant LHCGR expression since the protein profile of the supernantants from mock-transfected cultured cells was similar to that from DNA- transfected cells (data not shown).

### The release of recombinant LHCGR is mediated via microvesicles

The amount of total recombinant LHCGR on D4 and D5 in the supernatant was 4-12 fold higher than the corresponding total cellular proteins (Figure 3d-f), suggesting that the majority of protein synthesised on Days 1-3 was secreted on Days 4 and 5. The observation of specific secretion led us to hypothesise that the secretion might be active, involving sub-cellular organelles. To test this hypothesis, a three step centrifugation procedure was adopted whereby the highly clarified culture supernatant was subjected to ultracentrifugation in order to pellet any subcellular particles. As an alternative method, clarified culture supernatant was also concentrated by ultrafiltration (see Materials and Methods). Western blot analysis of the concentrated supernatant and the pellet following ultracentrifugation showed that only the pellet contained the LHCGR. Standard microscopy revealed subcellular particles with no detectable intact cells in the resuspended pellet (data not shown). Electron microscopy on the material from the pellet following ultracentrifugation revealed distinct subcellular particles. Based on the size of these particles (50-200 nm), the sLHCGR recombinants appear to have been released as components of these microvesicles (Figure 5d).

### Microvesicle-mediated release of sLHCGR from human placenta explants at early pregnancy

**Figure 6:**
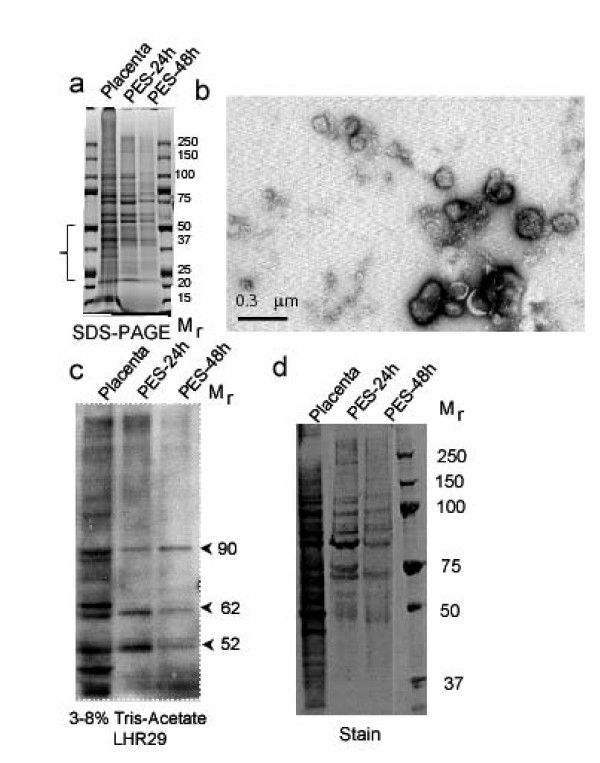
**The microvesicles from placenta explants contain both full-length and truncated LHCGR isoforms as well as selectively enriched proteins**. a), the placenta tissue extracts (Placenta) as well as proteins extracted from placenta explant microvesicles (PES) released into the culture medium at 24h (PES-24h) and 48h (PES-48h) were separated in 4-12% polyacrylamide SDS gels. The selective loss of proteins of M_r _50K-20K in the microvesicle fraction is indicated by a bracket; b), microvesicles purified by ultracentrifugation of the culture medium were examined by transmission electron microscopy; c) and d), microvesicle proteins include LHCGR as shown by c), Western blot probed with LHR29 monoclonal antibody and subsequently d), stained with coomassie blue.

To investigate whether release of sLHCGR is unique to cells transfected with LHCGR recombinants, dissected placental explants from early pregnancy (9 and 10 wks of gestation) were cultured up to 48h under identical conditions in serum-free medium as described above. Aliquots of culture medium were collected after 12h, 24h and 48h incubation. Analysis of the proteins from concentrated culture supernatant and the control placental extract (uncultured) by SDS-PAGE revealed quantitative and selective release of some proteins in the supernatant (Figure 6a). Electron microscopy analysis of the supernatant revealed the release of 50-300 nm vesicles from cultured placental explants (Figure 6b). Western blots performed on extracts from placenta and microvesicles with LHR29 monoclonal antibody against LHCGR (the epitope is mapped between 229-291 amino acids) showed that M_r _52K, 65K and 90K proteins were associated with microvesicles (Figure 6c and 6d). The absence or quantitative reduction of 37K, 65K, 85K and high molecular mass LHCGR isoforms in the explant supernatants compared to the control placenta (Figure 6c) confirmed selective association of LHCGR protein with microvesicles released from the placenta. These results provide evidence that the release of LHCGR from transfected cells via microvesicles is not unique and that natural, membrane-bound full-length LHCGR and truncated LHCGR variants are also released from placental tissues in this manner.

### Not all microvesicles from the placenta contain LHCGR

**Figure 7:**
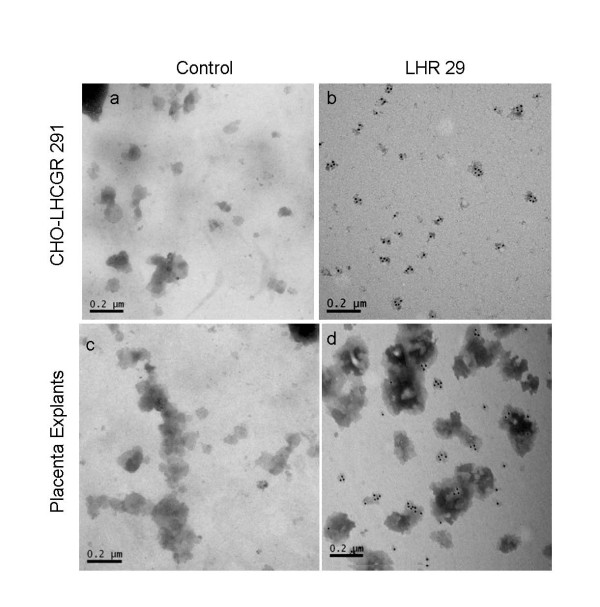
**Immuno-electron microscopy of microvesicles**. The purified microvesicles from LHCGR 291 cDNA transfected culture supernatant at day 4 (a & b) and placental explants PES-24h (c & d). In a & c (control), the microvesicles were reacted with non-specific mouse IgG and in b & d (LHR29), the primary antibody was LHR29 monoclonal antibody. The secondary antibody in each case was anti-mouse IgG gold-labeled antibody.

We sought another line of evidence to verify that the microvesicles released into the culture supernatant contain the LHCGR protein. This was achieved by immunostaining the microvesicles with LHCGR-specific (LHR29) primary antibody followed by anti-mouse IgG gold-labeled secondary antibody (Figure 7b &7d). As a control (Figure 7a &7c), mouse IgG was used as a primary antibody followed by anti-mouse IgG gold-labeled secondary antibody. Unlike the microvesicles derived from transfected CHO cell culture supernatant (Figure 7b), only 30-40% microvesicles released from human placenta (Figure 7d) were immunostained with LHCGR-specific antibody. The LHR29 monoclonal antibody recognizes an epitope that is between amino acid residues 227-289 of LHCGR at the N-terminus (see Materials & Methods). As an additional control the microvesicles from LHCGR 291 transfected cells (day 4) and placental explants (PES-24h) supernatants were first reacted with anti-FLAG mouse monoclonal primary antibody followed by anti-mouse IgG gold-labeled secondary antibody. As would be expected, only those microvesicles derived from transfected cells reacted with anti-FLAG antibody (data not shown). These experiments confirm that the LHCGR protein is integral to the microvesicles released into the culture supernatant for both recombinant transfectants and cultured placental explants and further show that only a fraction of the microvesicles released from the placenta contain LHCGR.

## Discussion

In this manuscript, we demonstrate that the secretion of soluble LHCGR from transfected cells as well as from placental tissues is independent of the size of the receptor and is mediated via microvesicles. Since microvesicles are also released from mock-transfected cells and only a portion of placental explant microvesicles stain positive for LHCGR, the mechanism of secretion is not specific to the LHCGR. The data presented here support previous work describing the abundant extracellular appearance of LH receptor and its natural variants lacking either transmembrane or intracellular domains, or both, following transfection with cDNA [11,12]. It is noteworthy that these authors speculated that the secreted receptor proteins could be packaged in membrane vesicles [12]. Additionally, while biochemical [9] and cell biological [10] studies demonstrated the release of LH receptor and hormone-receptor complex, our current data in conjunction with recent reports on the constitutive secretion of microvesicles from placenta throughout the human pregnancy [22], provide the possible mechanistic basis of the secretion of LHCGR from cells and tissues.

Questions have been raised regarding the conflicting results on LH receptor secretion described in the literature. In the light of the data presented in this manuscript, two explanations can be proposed that together might account for this discrepancy. Firstly, the methods used for *in vitro *expression and detection of the secreted receptor may be factors that affect experimental outcome. Secondly, the duration of experiments could be crucial. The reports of Tsai-Morris *et al*., and VuHai-LuuThi *et al*., [11,12] both describe clear-cut secretion of the soluble LH receptor proteins where experiments were carried out using transient transfection of cells (via calcium phosphate) and by assaying for the soluble LH receptor via ligand-binding assays and by Western blot. The method of transient expression [11,12] instead of stable cell lines [15,23], detection of the soluble receptor by immunoprecipitation [15] instead of ligand-binding assays might therefore have influenced the different experimental outcomes described by these groups. The duration of experiments could be pertinent to the failure to detect soluble LH receptor. Our data show that receptor expression, measured by Western blot on supernatants concentrated by ultrafiltration, following 72h of transfection, was barely detectable (Figure 3). Indeed, immunoprecipitation of 48h and 72h transfected supernatants with anti-FLAG monoclonal antibody failed to detect sLHCGR when the blots were probed with anti-LHCGR monoclonal antibody (data not shown). Therefore, a combination of the methods and biochemical conditions of *in vitro *expression of LH receptor, sensitivity of the assay detecting soluble receptor and the duration of the experiment together could explain the conflicting data in the literature.

Direct translocation of the cytoplasmic receptor isoforms across the plasma membrane or protease cleavage of membrane-bound extracellular domains are the mechanisms that have been proposed for circulating soluble receptors [24]. We demonstrate that LHCGR is released from transfected cells and placental explants packaged in microvesicles. Such secreted proteins released from tissues into the extracellular space fail to exhibit typical N-terminal secretory signal sequences as well as post-translational modifications necessary for traffic through Golgi/ER [25]. Human tissues under oxidative stress [26], following cellular activation such as p53 [27] and apoptosis release microvesicles ranging from relatively smaller (50-150nm) exosomes and plasma membrane-coated microparticles (100nm-1000nm), which represent fragments of cell membranes [28-32]. It should be noted however, that while the structural and immune-electron microscopic data indicate that released microvesicles could represent exosomes, future gradient density and biochemical analyses would be needed to confirm this.

The pleiotropic effects of microvesicles in inter-cellular communications have been intensely investigated in recent years. Given that microvesicles contain membrane proteins, mRNAs, miRNAs, growth factors, cytokine and chemokine receptors, it has been proposed that these circulating vesicles form a storage pool of bio-effectors or macromolecules for long-distance trans-cellular signaling/adhesion in endothelial modifications, angiogenesis, antigen presentation, immuno-suppression, differentiation and apoptosis in a variety of patho-physiological conditions including cancer [20,21,33-37]. Human placental syncytotrophoblasts constitutively secrete microvesicles throughout the pregnancy. Immune activation, immune-suppression by Fas-FasL-mediated apoptosis at the fetal-maternal interface and systemic vasculo-endothelial damage by microparticles and exosomes released from the placenta are emerging as modes of immune and vascular regulation in human pregnancies [22,38,39]. Our data showing that microvesicles released from the human placental explants contain full-length as well as truncated LHCGR raises the possibility that these vesicle-associated receptors, capable of binding LH and hCG hormones, may freely circulate in the blood. As such, the circulating membrane-bound LHCGR could cause reduced hormone bioactivity and may mediate abnormal activation of tissues through mobile receptor fusion. We propose that LHCGR-bearing microvesicles neutralize the natural ligands (LH or hCG) by forming soluble complexes which prevent the interactions of these hormones with tissue-specific target receptors. By altering hormone-mediated cellular activation of target tissues, the secreted, circulating sLHCGR may act as a physiological modulator of hormone function and/or as an inhibitor of hormone function that leads to pathology. This might explain the altered ratios of bioactive to immunoreactive LH or hCG that are frequently described in patients with hypogonadism [40], poor fertility [41-43], ovarian dysfunction [42,43], miscarriage [44-46] and other early pregnancy complications [47]. The possibility that circulating microvesicles bearing LHCGR might fuse with vasculo-endothelial or other cell types thereby making fused cells subject to LH/hCG activation, may be another level of complication that disrupts cell signaling. There is precedence for microvesicle-mediated transfer of the transferrin receptor [48], but whether the horizontal transfer of LHCGR occurs will require further investigation.

## Competing interests

Authors RH and PS have no competing interests. Authors AEC and SB are currently developing immunodiagnostics based on these observations.

## Authors' contributions

AEC carried out the transfection cell culture studies, helped with Western blotting, contributed to analysis and interpretation of results and participated in the draft of the manuscript. SB conceived and initiated the study, designed the cDNA constructs, carried out the glycosidase and placenta Western blotting experiments, designed and participated in the electron microscopy work, was instrumental in the analysis and interpretation of data, and drafted the manuscript. PS guided and helped with electron microscopy. HR participated in discussion, interpretation and manuscript preparation. All authors read and approved the final manuscript.
